# Non-secretory renin reduces oxidative stress and increases cardiomyoblast survival during glucose and oxygen deprivation

**DOI:** 10.1038/s41598-020-59216-8

**Published:** 2020-02-11

**Authors:** Heike Wanka, Philipp Lutze, Doreen Staar, Alexander Albers, Inga Bäumgen, Bianka Grunow, Jörg Peters

**Affiliations:** 1 Institute of Physiology, University Medicine Greifswald, 17475 Greifswald, Germany; 2 Leibniz Institute for Farm Animal Biology, Institute for Muscle Biology and Growth, Dummerstorf, Germany

**Keywords:** Heart failure, Molecular medicine

## Abstract

Although the renin-angiotensin system usually promotes oxidative stress and cell death, renin transcripts have been discovered, whose transcription product may be cardioprotective. These transcripts encode a non-secretory renin isoform that is localized in the cytosol and within mitochondria. Here we tested the hypotheses that cytosolic renin [ren(2-9)] expression promotes cell survival under hypoxia and glucose depletion by preserving the mitochondrial membrane potential (∆Ψ_m_) and mitigating the accumulation of ROS. To simulate ischemic insults, we exposed H9c2 cells to glucose deprivation, anoxia or to combined oxygen-glucose deprivation (OGD) for 24 hours and determined renin expression. Furthermore, H9c2 cells transfected with the empty pIRES vector (pIRES cells) or ren(2-9) cDNA-containing vector [ren(2-9) cells] were analyzed for cell death, ∆Ψ_m_, ATP levels, accumulation of ROS, and cytosolic Ca^2+^ content. In pIRES cells, expression of ren(1A-9) was stimulated under all three ischemia-related conditions. After OGD, the cells lost their ∆Ψ_m_ and exhibited enhanced ROS accumulation, increased cytosolic Ca^2+^ levels, decreased ATP levels as well as increased cell death. In contrast, ren(2-9) cells were markedly protected from these effects. Ren(2-9) appears to represent a protective response to OGD by reducing ROS generation and preserving mitochondrial functions. Therefore, it is a promising new target for the prevention of ischemia-induced myocardial damage.

## Introduction

Myocardial infarction is a major cause of death worldwide. Given the high incidence of myocardial infarction and the associated cardiac injury, developing novel strategies and countermeasures to protect the heart against acute and especially ischemia/reperfusion-induced damage is of great interest.

Renin is known as secretory glycoprotein that generates angiotensin (ANG) I from angiotensinogen. ANG I is further cleaved to ANG II by the angiotensin-converting enzyme. ANG II increases blood pressure as well as salt- and water reabsorption. Furthermore, ANG II enhances oxidative stress, exerts pro-inflammatory effects and induces apoptotic and necrotic cell death, particularly in the heart and the kidney. Correspondingly, inhibitors of the renin-angiotensin system (RAS) are among the most potent drugs in the treatment of hypertension and cardiac failure, markedly increasing the life span of patients^1^.

Alternative renin transcripts, termed exon1A renin, exon(2-9)renin, renin-b or renin-c, have been identified in rats and mice^2,3^ as well as in transgenic mice expressing a human renin gene construct^4^. In the rat heart, exon(1A-9) renin transcription is under the control of an alternative promoter located in intron 1^5^. In cardiac cells, this promoter is stimulated by glucose depletion in a serum response factor-dependent manner^5^. Furthermore, exon(1A-9) renin mRNA abundance increased markedly after myocardial infarction *in vivo*^6^. Due to the absence of the signal for a co-translational transport to the endoplasmatic reticulum, encoded by exon1, all alternative renin transcripts are translated at free ribosomes into a truncated prorenin^2–4^. The protein is found in the cytosol as well as within mitochondria^2,7,8^.

Ren(2-9) transfected H9c2 cells exposed to glucose starvation are protected from necrotic cell death^9^. Although basal rates of apoptosis were increased in these cells^8^, hearts of transgenic rats overexpressing ren(2-9) remained functionally and morphologically unchanged. The increased basal apoptosis rate is explained by non-mitochondrial ATP generation despite presence of enough oxygen. However, under depletion conditions the ren(2-9)cells are already adapted to the lack of oxygen (warburg effect). Furthermore, the hearts of these transgenic rats analyzed *ex vivo* in the Langendorff preparation were more resistant against ischemia-induced injury^9^. Therefore, we speculated that under ischemia-related conditions such as glucose depletion, hypoxia or both together (OGD) ren(2-9) exerts anti-necrotic and anti-apoptotic effects by preserving the mitochondrial membrane potential (∆Ψ_m_) and mitigating the accumulation of reactive oxygen species (ROS).

## Results

### Exon(1A-9)renin transcript levels increase under glucose and/or oxygen depletion in pIRES and ren(2-9) cells

For these experiments, we generated a cell line overexpressing ren(2-9) mRNA about 10-fold [ren(2-9) cells]. We excluded the so-called “exon1A”, since it is non-coding and may have regulatory functions that we thus avoided. The degree of overexpression was similar to the degree of endogenous exon(1A-9)renin expression overexpression found after glucose depletion or anoxia.

**Figure 1:**
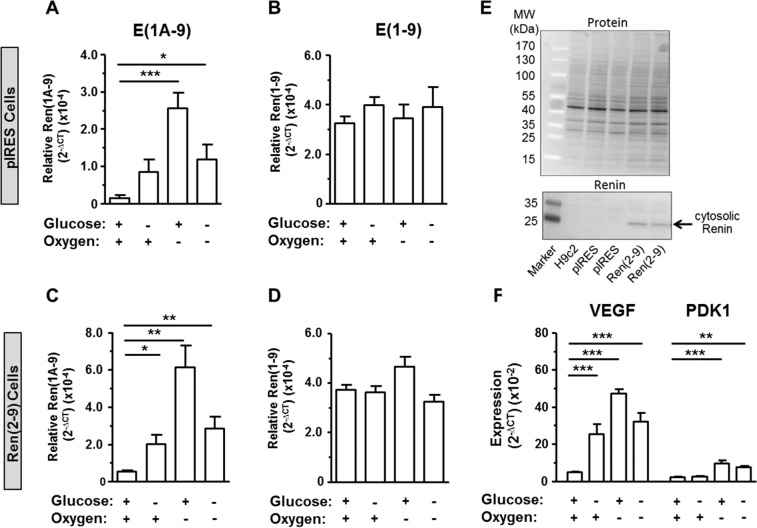
Expression of renin transcripts in transfected pIRES and ren(2-9) cells. Cardiac pIRES controls (empty vector-transfected cells) and ren(2-9)-overexpressing cells [Ren(2-9)] were exposed to control conditions, glucose (Glc) depletion alone, anoxia alone, or the combination of oxygen and glucose depletion (OGD) for 24 hours. (**A**–**D**) Renin transcript levels or (**F**) hypoxia-relevant genes normalized to the housekeeper YWHAZ were quantified by RT-PCR. (**E**) Renin protein of pIRES controls and ren(2-9) cells normalized to the protein content was detected by Western Blot. The data represent mean ± SEM values of 5-7 independent experiments or representative Western blots. *p < 0.05, ***p < 0.001 (Anova, Kruskis-Wallis (**A**–**D**) or Bonferroni (**E**)), E(1A-9): exon(1A-9)renin, E(1-9): exon(1-9)renin.

In pIRES control cells as well as in ren(2-9) cells, exon(1-9)renin mRNA abundances were neither affected by glucose depletion or anoxia alone, nor by OGD (Fig. 1B,D). In contrast, exon(1A-9)renin mRNA expression increased in both cell lines after exposure to glucose depletion and anoxia as well as after OGD (Fig. 1A,C). We already demonstrated that glucose depletion increased ren(2-9) protein in previous studies. We here further confirm that the ren(2-9) protein level was increased in ren(2-9) transfected cells already prior to the depletion conditions (Fig. 1E).

To verify the success of experimentally induced anoxia in our setting, we analyzed the expression levels of the HIF-regulated genes vascular endothelial growth factor (VEGF) and pyruvate dehydrogenase kinase 1 (PDK1) (Fig. 1F). The expression of VEGF in H9c2 cells was markedly upregulated by all three ischemia-related conditions. PDK1 transcript levels also increased significantly in anoxia and in OGD-exposed cells, respectively, but not by glucose depletion alone.

### Ren(2-9) protects from ischemia-induced apoptosis and necrosis

**Figure 2:**
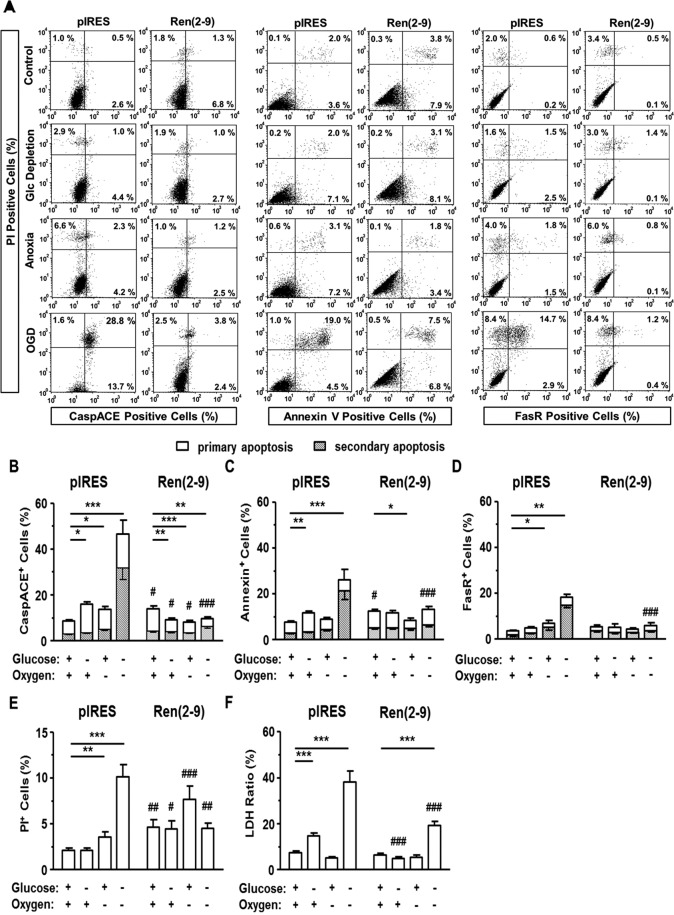
Ren(2-9) protects H9c2 cells from apoptotic and necrotic death induced by ischemia-related conditions. Cardiac H9c2 pIRES controls (empty vector-transfected cells) and ren(2-9)-overexpressing cells [Ren(2-9)] were exposed to control conditions, glucose (Glc) depletion alone, anoxia alone, or the combination of oxygen and glucose depletion (OGD) for 24 hours. (**A**) Representative histograms of apoptotic cells labelled with apoptosis-specific markers and propidium iodide (PI). Apoptosis rate was quantified by the percentage of (**B**) CaspACE^+^, (**C)** Annexin V^+^ and (**D**) Fas receptor^+^ cells (n = 8–9, each). Cells were additionally labelled with PI to differentiate between early apoptosis (PI^−^) and late apoptosis (PI^+^) (grey shaded). Necrosis rate was determined (**E**) by PI labelling (early necrosis, PI^+^ apoptosis^-^ cells, n = 9) and (**F**) by the percentage ratio between released LDH and LDH content using the cytotoxicity detection kit (n = 10). Data represent mean ± SEM values. *p < 0.05, **p < 0.01, and ***p < 0.001 vs. basal conditions with glucose and oxygen; ^#^p < 0.05, ^##^p < 0.01, and ^###^p < 0.001 vs. pIRES controls.

We next asked whether the coding part of the ren(2-9) transcript can protect cardiac H9c2 cells from early and late apoptosis as well as necrosis. Using propidium iodide (PI) labelling, we differentiated between early (PI^−^) and late (PI^+^) apoptotic states. We confirmed former data that under basal conditions, ren(2-9) cells exhibited higher apoptosis rates, especially early apoptosis, than vector-transfected pIRES control cells (Fig. 2) with respect to caspase activation (Fig. 2A,B) and Annexin V labelling (Fig. 2A,C). Fas receptor (FasR) expression remained unchanged between controls and ren(2-9) cells (Fig. 2A,D).

In contrast to the basal situation, the rate of early apoptosis was markedly lower in ren(2-9) cells than in pIRES control cells after glucose depletion. Whereas glucose depletion markedly increased early apoptosis in pIRES control cells without influencing late apoptosis rate, glucose depletion even led to a significant decrease in the rate of PI^−^ CaspACE^+^ ren(2-9) (Fig. 2A,B–D). Fas receptor expression again remained unchanged in both cell lines (Fig. 2A,D).

Anoxia alone had no effect on the percentages of CaspACE^+^ or Annexin V^+^ pIRES control cells, whereas the percentage of PI^+^ FasR^+^ cells increased (Fig. 2A–C). Overexpression of ren(2-9) protected the cells from anoxia-induced apoptosis as well as from apoptosis.

Exposure of pIRES cells to OGD substantially stimulated early and late apoptosis rates (Fig. 2A,B–D). In contrast to pIRES control cells, ren(2-9) overexpressing cells were entirely protected from OGD-induced early and late apoptosis (Fig. 2A,B–D). In fact, in ren(2-9) cells the percentages of Annexin V^+^ and FasR^+^ cells remained essentially unaffected after OGD and were significantly less than in pIRES cells. The percentage of PI^−^ CaspACE^+^ ren(2-9) cells even decreased (Fig. 2A–C).

With respect to early necrotic cells, the percentage of PI^+^ apoptosis^−^ pIRES cells was unaltered by glucose depletion but increased after anoxia and OGD, respectively (Fig. 2A,E). Compared to pIRES cells, ren(2-9) cells showed a higher percentage of PI^+^ apoptosis^−^ cells under basal conditions and a decreased percentage after OGD. The basal level of early necrotic ren(2-9) cells was not significantly affected by any of our ischemia-related interventions (Fig. 2A,E).

Late necrosis rates, expressed as the percentaged ratio between LDH release and LDH content, were not significantly different between the two cell lines under basal conditions (Fig. 2F). In pIRES cells, glucose depletion alone and in combination with anoxia increased necrosis rates, while anoxia alone had no significant effect. In ren(2-9) cells neither glucose depletion alone nor anoxia alone significantly affected necrosis rates, while OGD increased the necrosis rate. Notably, the OGD-induced increase in necrosis rates was significantly lower in ren(2-9) cells than in pIRES cells. In summary we here observed a prominent protective anti-necrotic and anti-apoptotic effect of ren(2-9) overexpression under ischemia-related conditions, particularly under OGD.

### Ren(2-9) protects from OGD-induced ROS accumulation

**Figure 3:**
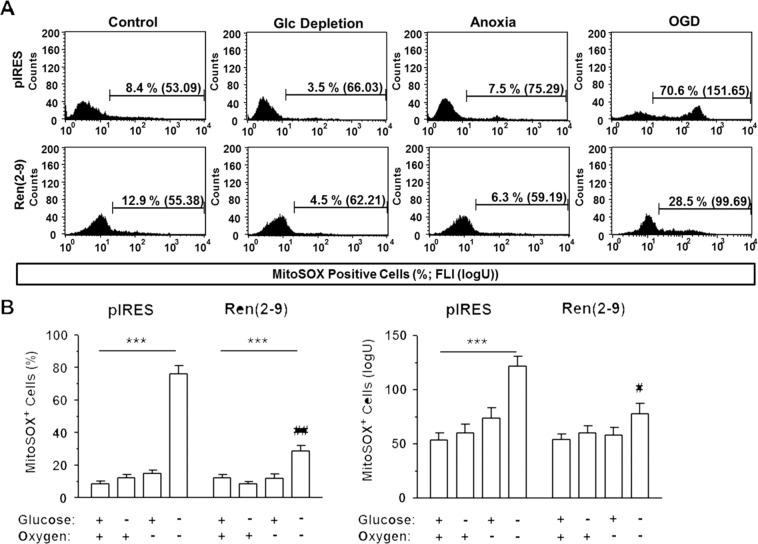
Ren(2-9) protects H9c2 cells from accumulation of mitochondrial superoxides induced by ischemia-related conditions. Cardiac pIRES controls (empty vector-transfected cells) and ren(2-9)-overexpressing cells [Ren(2-9)] were exposed to control conditions, glucose (Glc) depletion alone, anoxia alone or the combination of oxygen and glucose deprivation (OGD) for 24 hours. Afterwards, cells were incubated with MitoSOX fluorophore to detect mitochondrially localized superoxides. (**A**) Representative histograms and (**B**) analyses of percentage and mean fluorescence intensity of MitoSOX^+^ cells. Data represent mean ± SEM values of 9-10 experiments. ***p < 0.001 vs. basal conditions with glucose and oxygen; ^#^p < 0.05, ^##^p < 0.01 vs. pIRES controls.

Because one reason for cell death during ischemia is the imbalance between the generation and degradation of reactive oxygen species (ROS), we next investigated the influence of ren(2-9) on ROS levels in mitochondria and cytosol. Using the MitoSOX fluorophore, we quantified the fraction of MitoSOX^+^ cells and their mean fluorescence intensity (FLI), the quantitative indicator of mitochondrial superoxide accumulation (Fig. 3A,B). Under control conditions, the percentage of MitoSOX^+^ cells as well as their mean FLI showed similar levels in both cell lines. Neither glucose starvation alone nor anoxia alone changed the percentage or the FLI of MitoSOX^+^ cells in pIRES controls and ren(2-9) cells, respectively. Yet, OGD resulted in an increase of mitochondrial superoxides, which was much less pronounced in ren(2-9) cells than in pIRES controls.

**Figure 4:**
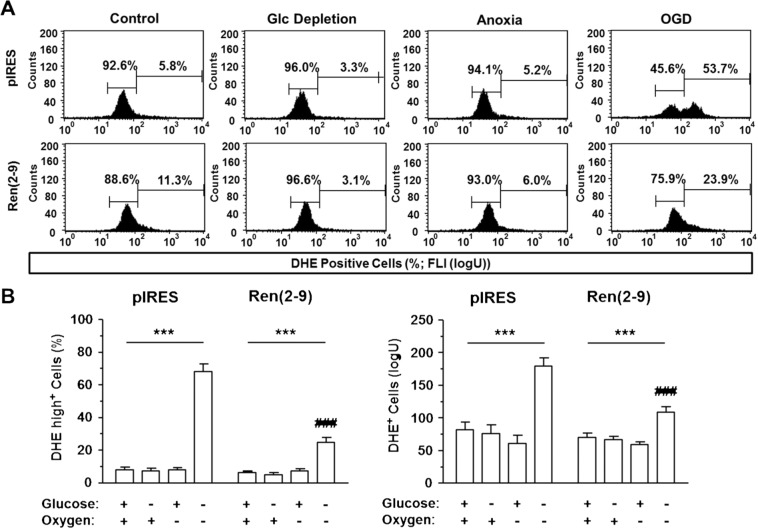
Ren(2-9) protects H9c2 cells from accumulation of cytosolic reactive oxygen species induced by ischemia-related conditions. Cardiac pIRES controls (empty vector-transfected cells) and ren(2-9)-overexpressing cells [Ren(2-9)] were exposed to control conditions, glucose (Glc) depletion alone, anoxia alone or the combination of oxygen and glucose deprivation (OGD) for 24 hours. Afterwards, cells were incubated with dihydroethidium (DHE) to detect reactive oxygen species in the cytosol. (**A**) Representative histograms and (**B**) analyses of percentage (left panel) and mean fluorescence intensity of DHE^+^ cells (right panel). Data represent mean ± SEM values of 7-10 experiments. ***p < 0.001 vs. basal control with glucose and oxygen; ^###^p < 0.001 vs. pIRES cells.

Cytosolically localized ROS were quantified by dihydroethidium (DHE). Under basal conditions, nearly all cells were DHE^+^ (Fig. 4A). Additionally, few cells of each cell line, the DHE high^+^ cells, showed an increased FLI (disturbed ROS management) (Fig. 4A). Thus, we quantified both the percentage of cells with an intermediate FLI and that with an enhanced FLI. Using this scheme, the percentages and the mean FLI of DHE high^+^ cells were similar in both cell lines (Fig. 4B). Neither glucose starvation alone nor anoxia alone significantly affected the percentages of DHE high^+^ cells or their mean FLI. Yet, the combination of both stimuli increased cytosolic ROS levels in pIRES controls but not in ren(2-9) cells (Fig. 4B). In OGD-treated ren(2-9) cells, the percentage of DHE high^+^ cells increased only fourfold (Fig. 4B).

### Ren(2-9) reduces ischemia-induced disruption of mitochondrial membrane potential and permeability

**Figure 5:**
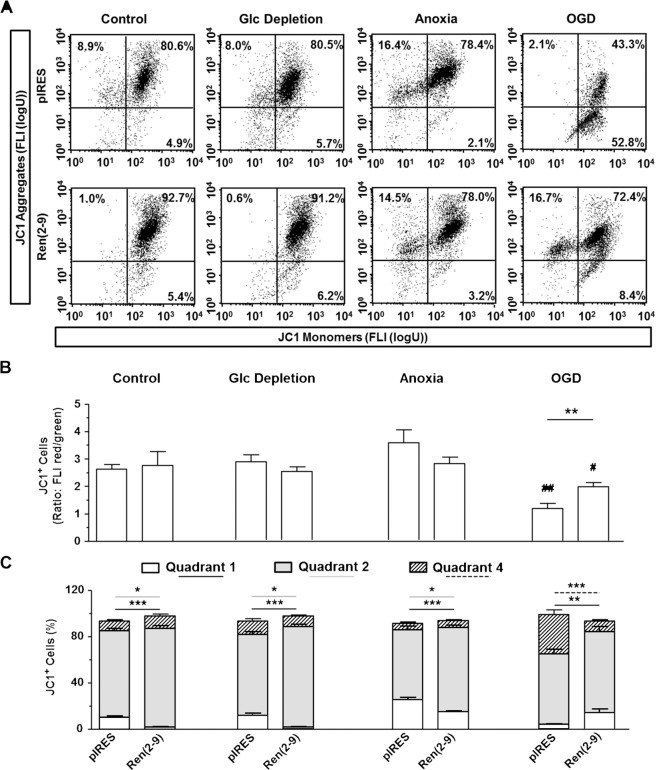
Ren(2-9) protects H9c2 cells from disruption of mitochondrial membrane potential induced by ischemia-related conditions. Cardiac pIRES controls (empty vector-transfected cells) and ren(2-9)-overexpressing cells [Ren(2-9)] were exposed to control conditions, glucose (Glc) depletion alone, anoxia alone or the combination of oxygen and glucose deprivation (OGD) for 24 hours. Afterwards, cells were incubated with JC1 dye to detect the mitochondrial membrane potential (∆Ψ_m_). (**A**) Representative histograms of JC1^+^ cells separated in different quadrants according to the generation of JC1 aggregates (red fluorescence) or JC1 monomers (green fluorescence). (**B**) Analysis of the ratio of red/green fluorescence intensity (FLI) representing the ∆Ψ_m_. (**C)** Distribution of JC1^+^ cells within the different quadrants according to their red or green fluorescence. Localization of quadrant 1: upper left, quadrant 2: upper right, quadrant 4: bottom right. Data show mean ± SEM values of 8 experiments. *p < 0.05, **p < 0.01, ***p < 0.001 vs. pIRES cells, ^#^p < 0.05, ^##^p < 0.01, ^###^p < 0.001 vs. basal controls.

The height of the mitochondrial inner membrane potential (∆Ψ_m_) depends on inner membrane integrity and can be impaired by ischemia. We used the fluorescence dye JC1 to assess ∆Ψ_m_. The ratio between red (JC1 aggregates, quadrants 2 and 3; high ∆Ψ_m_) and green FLI (JC1 monomers, quadrant 4; low ∆Ψ_m_) can be used as a proxy for ∆Ψ_m_ (Fig. 5A,B). We additionally determined the distribution of cells among the three quadrants (Fig. 5A,C). Under basal conditions and glucose starvation, ∆Ψ_m,_ expressed as red to green FLI ratio, was similar between pIRES controls and ren(2-9) cells. However, the distribution of cells within the quadrants differed between both lines, especially the percentage of cells in quadrants 1 and 2 (Fig. 5C). Under basal conditions, 10.1 ± 1.4%, 75.0 ± 2.2%, and 8.3 ± 1.4% of JC1^+^ pIRES cells were located in quadrants 1, 2, and 4, respectively, while only 1.6 ± 0.5% of JC1^+^ ren(2-9) cells were found in quadrant 1, 85.6 ± 2.4% and 10.8 ± 1.4% in quadrants 2 and 4, respectively. Glucose depletion alone neither modified ∆Ψ_m_ nor the cellular distribution among the quadrants. Anoxia alone increased ∆Ψ_m_ in pIRES cells, but did not significantly alter ∆Ψ_m_ in ren(2-9) cells. Furthermore, the fraction of JC1^+^ pIRES cells increased in quadrant 1 and decreased in quadrant 2, whereas anoxia had no statistically significant effects on the distribution of JC1^+^ ren(2-9) cells among the quadrants.

OGD decreased the FLI red to green ratio in pIRES, indicating a collapse of ∆Ψ_m_. This decrease was due to a shift of JC1^+^ cells from quadrant 2 with high ∆Ψ_m_ to quadrant 4 with low ∆Ψ_m_. OGD also decreased the FLI red to green ratio in ren(2-9) cells, albeit to a significantly lesser extent than in pIRES cells. This decrease was also associated with a shift of JC1^+^ cells from quadrant 2 to quadrant 1.

**Figure 6:**
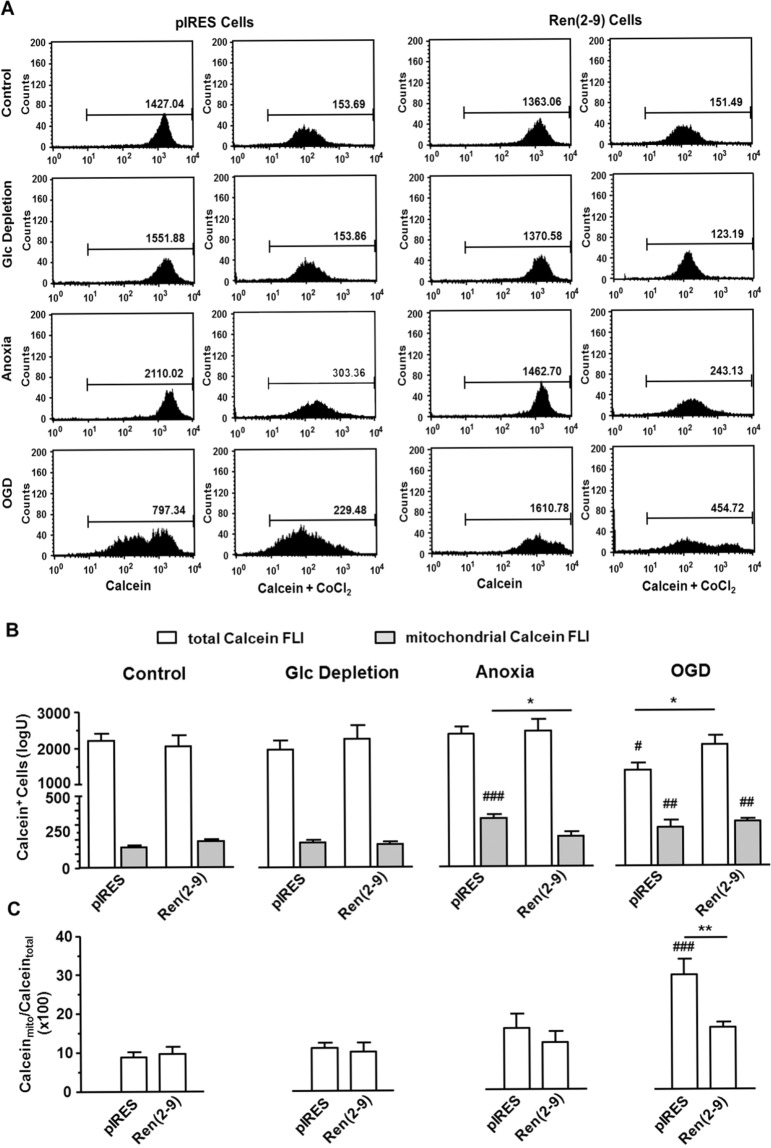
Ren(2-9) protects H9c2 cells from mitochondrial calcein overload induced by ischemia-related conditions. Cardiac pIRES controls (empty vector-transfected cells) and ren(2-9)-overexpressing cells [Ren(2-9)] were exposed to control conditions, glucose (Glc) depletion alone, anoxia alone or the combination of oxygen and glucose deprivation (OGD) for 24 hours. Afterwards, cells were incubated with calcein-AM or calcein-AM plus CoCl_2_ to monitor the distribution of calcein within all subcellular compartments combined vs. mitochondria alone. (**A**) Representative histograms of total vs. mitochondrial calcein fluorescence intensities (FLI). (**B**) Analyses of total (white columns) vs. mitochondrial calcein FLI (grey columns). (**C**) Ratio of mitochondrial to total calcein FLI representing mitochondrial permeability transition. Data represent mean ± SEM values of 8 experiments. *p < 0.05, **p < 0.01 vs. pIRES cells, ^##^p < 0.01, ^###^p < 0.001 vs. basal control.

Since the reduction of ∆Ψ_m_ may have been caused by an opening of the mitochondrial permeability transition pore (mPTP), we next examined the effects of ischemia-related conditions on the mPTP using the calcein-CoCl_2_ method^10^. Calcein-AM is a membrane permeable fluorophore that diffuses into all subcellular comportments including mitochondria (total calcein fluorescence) (Fig. 6A). Upon Calcein-AM uptake, cellular esterases cut off the AM group and calcein binds to Ca^2+^, resulting in acquired green fluorescence. Addition of CoCl_2_ quenches the calcein signal in all subcellular compartments except the mitochondrial matrix (mitochondrial calcein fluorescence) (Fig. 6A). Under control conditions and after glucose starvation, total calcein fluorescences were similar in pIRES and ren(2-9) cells, indicating similar calcein loading (Fig. 6A,B). Mitochondrial calcein signals were about 10-fold lower than total calcein fluorescence in both cell lines and remained unchanged by glucose starvation (Fig. 6B). Anoxia induced a significant increase of mitochondrial calcein fluorescence in pIRES cells, but not in ren(2-9) cells, reflecting an overload of mitochondrial calcein in pIRES cells (Fig. 6B). Despite this increase, the percentaged ratio of mitochondrial to total calcein fluorescence remained unchanged in pIRES cells (Fig. 6C).

OGD markedly increased the mitochondrial-to-total calcein fluorescence ratio due to a significant decrease of the total and an increase of the mitochondrial calcein signal in pIRES cells. As demonstrated in the dot plot histograms (Fig. 6A), the mean decline in the total calcein fluorescence after OGD can be explained by a partial shift to lower FLIs in around 50% of the pIRES cells. In ren(2-9) cells, we observed also an increase of the mitochondrial calcein signal, but total calcein fluorescence and the ratio between both signals were unchanged.

### Ren(2-9) prevents the OGD-induced increase of cytosolic Ca^2+^

**Figure 7:**
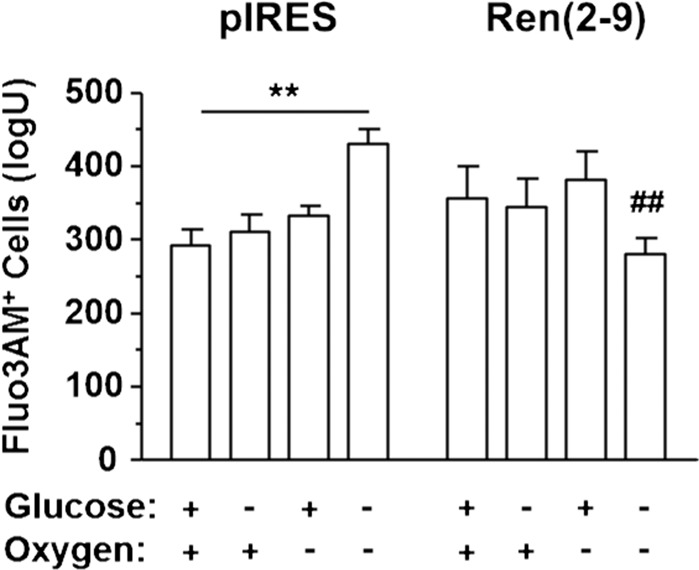
Ren(2-9) protects H9c2 cells from OGD-induced cytosolic Ca^2+^ overload. Cardiac pIRES controls (empty vector-transfected cells) and ren(2-9)-overexpressing cells [Ren(2-9)] were exposed to control conditions, glucose depletion, anoxia or the combination of oxygen and glucose deprivation (OGD) for 24 hours. Afterwards, cells were incubated with the fluorophore Fluo3AM to detect cytosolic free Ca^2+^ levels via analysis of fluorescence intensity. Data show mean ± SEM values of 10 experiments. **p < 0.01 basal vs. control, ^##^p < 0.01 vs. pIRES cells.

One component influencing ∆Ψ_m_ and cell death is Ca^2+^ overload of the cell^11^. Therefore, we used the Ca^2+^-sensitive fluorophore Fluo3-AM to monitor the fluorescence intensity of Fluo3 that reflects free cytosolic Ca^2+^ (Fig. 7). In pIRES controls the Ca^2+^ content remained unchanged after glucose depletion or anoxia alone, while OGD significantly increased the Ca^2+^ level. Again, in ren(2-9) cells neither glucose depletion alone nor anoxia alone significantly affected the cytosolic Ca^2+^ level. In contrast to its effect in pIRES cells, OGD did not significantly affect the cytosolic Ca^2+^ level in ren(2-9) cells. In fact, Fluo3 FLI after OGD was significantly lower in ren(2-9) cells than in pIRES controls, suggesting that cyto-renin overexpression completely prevented the OGD-induced increase in cytosolic Ca^2+^.

### Ren(2-9) attenuates OGD induced decreases of ATP levels

**Figure 8:**
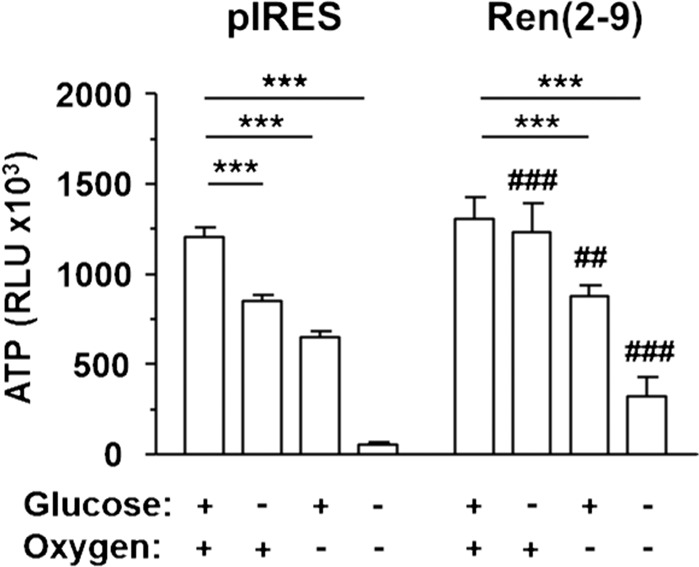
Ren(2-9) limits decrease of ATP levels induced by ischemia-related conditions. Cardiac pIRES controls (empty vector transfected cells) and cyto-renin overexpressing cells [Ren(2-9)] were exposed to control conditions, glucose depletion alone, anoxia alone or the combination of glucose and oxygen deprivation (OGD) for 24 hours. Afterwards, cellular ATP levels were determined by the CellTiter Glo Cell Viability Assay. Data show mean ± SEM values of 6 experiments. ***p < 0.001 vs. basal control, ^##^p < 0.01, ^###^p < 0.001 vs. pIRES.

The cellular ATP level is the most important parameter governing the fate of the cell. Using the CellTiter-Glo Viability assay, we found a stress-dependent decrease of ATP levels in pIRES cells (Fig. 8). Glucose depletion alone already resulted in a significant reduction of the ATP content. Anoxia alone decreased the ATP level even further, while OGD was associated with a severe loss of cellular ATP. These harmful effects were attenuated by ren(2-9). Indeed, glucose depletion alone did not affect the ATP level of ren(2-9) cells. In oxygen-deprived ren(2-9) cells, the ATP loss was less severe than in oxygen-deprived pIRES cells. Furthermore, ren(2-9) cells exposed to OGD maintained the ATP level explaining the observed protective effects on cell survival and mitochondrial functions.

## Discussion

The present study demonstrates that overexpression of ren(2-9) can minimize apoptotic and necrotic cell death under ischemia-related conditions, especially under OGD. The protective effects of ren(2-9) can be explained by the preservation of mitochondrial structure and ATP levels, the reduction of superoxide accumulation in mitochondria and cytosol, as well as the avoidance of a cytosolic Ca^2+^ overload.

It is well accepted that mitochondria play a central role in cellular survival during ischemia because they are intimately involved in the control of cellular energy metabolism and operate as gatekeepers of death pathways^12,13^. Maintaining mitochondrial function is, therefore, an effective strategy to attenuate ischemia-induced injury of cardiac cells. Based on this concept, we hypothesized that the cardioprotective effects of ren(2-9) observed in transgenic rat hearts and H9c2 cells overexpressing ren(2-9)^9^ may be at least partially attributed to the maintenance of mitochondrial function during such conditions. The present data confirm the concept that the death pathways, i.e. apoptosis or necrosis, depend on the intensity and nature of the ischemic stress components, namely glucose starvation alone, anoxia alone, or OGD. Apoptosis can be divided into either intrinsically or extrinsically induced forms, depending on the initial event of the death signal cascade or into early and late apoptosis depending on the maintenance or the loss of cell membrane integrity.

After glucose depletion, pIRES cell death was caused by mild necrosis and by early intrinsic apoptosis as documented by the increased percentages of PI^-^ CaspACE^+^ and PI^-^ Annexin V^+^ cells. In this context, phosphatidylserine externalization from the inner to the outer membrane site as requirement for the binding of Annexin V dye has been identified as an early and prominent feature of mitochondrially-mediated apoptosis^14,15^. Therefore, we estimated the increased percentages of PI^-^ CaspACE^+^ and PI^-^ Annexin V^+^ cells following glucose depletion as indicator of an intrinsic, mitochondrially-mediated apoptosis path where phosphatidylserine externalization is accompanied by the activation of caspases.

After anoxia, in contrast, the percentages of PI^-^ CaspACE^+^ and Fas receptor^+^ pIRES control cells increased. These results point towards the activation of a primary extrinsic pathway, in which Fas receptor expression leads to the activation of caspases. Tanaka *et al*.^16^ documented that the enhanced Fas expression in rat primary neonatal cardiomyocytes after hypoxia could be due to the enhanced production of lactate followed by acidification of the media. Such effects also occurred in our system, where pIRES cells exposed to oxygen deprivation showed a doubling of extracellular lactate accumulation (data not shown). However, other explanations cannot be ruled out, including the expression of TNF, IL-1 or IL-6 that upregulate the Fas (CD95) system^17–19^.

Under OGD, the most stringent condition that limits both mitochondrial respiratory chain and anaerobic glycolysis, ATP is more deeply exhausted than under the milder stress of glucose depletion or anoxia alone. Thus, in pIRES cells OGD reduced the ATP content to only 5% of its basal level implicating that ATP-dependent apoptotic pathways must give way to necrotic cell death^20^. Considering the strong effects on necrosis described below under OGD, however, the OGD-induced decrease of ATP not only reflects the cellular ATP-depletion but also a reduction of cell number per well. OGD caused the collapse of pIRES cell viability leading to severe necrosis but also to late extrinsically and intrinsically triggered apoptosis. This is consistent with other studies where OGD induced both necrosis and apoptosis in cardiac cells^21,22^. Concerning pIRES cell apoptosis during OGD, the observed phosphatidylserine externalization was accompanied by and therefore likely caused by elevated levels of intracellular Ca^2+^, ROS generation and/or ATP depletion. All of these factors are described to inactivate the aminophospholipid translocase, which is necessary for the maintenance of asymmetrical distribution of phospholipids in the plasma membrane to adhere phosphatidylserine at the inner side^23^. In analogy, excessive oxidative stress and increased intracellular Ca^2+^ levels are responsible for cardiac death but especially during a reperfusion period following ischemia^24,25^. In this regard, we have to consider that most of our functional assays were done with a time-delay and during normoxic conditions, which may mimic a short reperfusion period.

A key component required for ATP synthesis is the electrochemical proton gradient across the inner mitochondrial membrane. The gradient depends on the ∆Ψ_m_ and is maintained by the state of the mPTP. The mPTP is a large-conductance channel with an oscillatory on/off state under physiological conditions. Excessive ROS generation and increased levels of Ca^2+^ within the mitochondrial matrix both trigger mPTP opening^24,26,27^. Excessive mPTP opening then leads to the disruption of the ∆Ψ_m_, increase of ROS generation, arrest of ATP synthesis and the entry of solutes and water into the mitochondrial matrix. Due to the resulting swelling of the matrix and rupture of the outer mitochondrial membrane, pro-apoptotic proteins are released from the intermembrane space and cause mitochondrially mediated cell death through apoptosis and necrosis^28,29^. OGD-subjected pIRES cells clearly showed a collapsed ∆Ψ_m_ accompanied by the accumulation of mitochondrial superoxides as evidenced by increased MitoSOX FLI. Besides the increase in MitoSOX FLI, the labelling of nearly 80% of the cells by MitoSOX illustrates the dysregulation of the ROS management. As a limitation of our study, we did not monitor the mitochondrial Ca^2+^ level directly by Rhod2. However, the calcein/CoCl_2_ method allows an indirect estimation because calcein binding to free Ca^2+^ results in the appearance of calcein fluorescence. The increased calcein signal within the mitochondria of OGD-exposed pIRES cells can thus be taken as a sign of an enhanced mitochondrial Ca^2+^ level. The mitochondrial Ca^2+^ overload leads then to the opening of the mPTP and the disruption of the ∆Ψ_m_. In summary, ROS, Ca^2+^ and mitochondrial injury may interact with and stimulate each other to synergistically regulate cell death during OGD.

Ischaemic preconditioning (IPC) is described as endogenous mechanism during brief and repetitive periods of transient ischemia-reperfusion to render the heart more resistant to injury from a subsequent longer ischaemic insult^30^. Thus, Yellon *et al*.^31^ showed that in patients with coronary artery bypass surgery only the heart of those patients showed a preservation of the ATP level and a better outcome which were subjected to preconditioning by two periods of short breaks of blood flow followed by reperfusion. Critical target of IPC is the mPTP whose excessive opening in the first few minutes of reperfusion induces cardiomyocyte death. Key inducers of mPTP opening are ATP depletion, excessive generation of ROS, mitochondrial Ca^2+^ overload and changes of intracellular pH^32^. Therefore, preserving ATP levels, attenuation of oxidative stress and mitochondrial Ca^2+^ overload, and delayed correction of ischemia-induced intracellular acidosis are integrated in the IPC-mediated inhibition of mPTP opening^33,34^. All of these protective events, preserved ATP levels, reduced ROS generation and Ca^2+^ overload, inhibition of mPTP opening and lastly reduced apoptosis and necrosis were indeed seen in OGD-exposed ren(2-9) cells indicating a preconditioning potential of ren(2-9). Furthermore, previous data demonstrate metabolic changes in ren(2-9) cells that are associated with an enhanced stress tolerance due to the switch to more aerobic glycolysis accompanied by the acidification of the culture medium and an altered bioenergetic profile (warburg effect)^35^.

The data of the present study demonstrate that ren(2-9) overexpression is protective under OGD. We cannot proof yet that the increase of endogenous ren(1A-9) exerts the same protective effect. It may be, however, of advantage that in our model of overexpression the elevated level of ren(2-9) is present already prior to OGD, so that precious time is saved. Although it was not the intention of the present study to unravel the mechanism of action, some speculations may be allowed here. First of all, cytosolic renin may contribute to angiotensin generation within the cytosol or even within mitochondria, although this matter is discussed controvers (for review see:^36–38^). Data from diabetic animals and patients demonstrate an increased intracellular ANG II level which is associated with enhanced oxidative stress, fibrosis, and cardiac cell apoptosis and necrosis^39^. These harmful intracellular ANG II effects are in contrary to our protective data. Mitochondrial and nuclear angiotensin receptors have been detected and angiotensin II indeed exerts effects on isolated mitochondria^40^) and nuclei^41^. Again, the effects of angiotensin II were always harmful unless AT1 receptors were blocked or absent. Nevertheless, the existence of renin transcripts encoding for cytosolic renin provides at least an important requirement for cytosolic or mitochondrial generation of angiotensins, which may be protective via AT2 receptors^40,41^. Thus, intracellular angiotensin generation may still be considered.

Another mechanism possibly taking part in the protective effects may be the interaction between cytosolic renin and the putative renin binding protein also known as N-acetyl-D-glucosamine 2-epimerase (NAGE)^42^. Renin-mediated inhibition of NAGE activity occurs especially under ischemic conditions where high-energy nucleotides such as ATP are depleted^43^. By regulating the availability of N-acetyl-glucosamine, the substrate for O-linked N-acetyl-glycosylation, cytosolic renin may influence the post-translational modification of cellular proteins. Indeed, O-glycosylation is known to mediate cardioprotection but also cardiovascular dysfunctions^44^. However, at present we cannot provide unequivocal data demonstrating interactions between cytosolic renin and NAGE in our models.

In summary, we present a new prominent protective effect of non-secretory renin which is manifested under ischemia-relevant conditions and may act in a preconditioning-like manner. We are aware of the fact that H9c2 cells do not reflect the situation in differentiated cardiomyocytes of adult heart, but just the situation of immature precursur cells. Nevertheless, the beneficial effects of ren(2-9) may well be present in adult human cardiomyocytes as well and then be helpful for the prevention of cardiac damage during myocardial infarction.

## Materials and Methods

### Cell culture

The H9c2 cell line obtained from American Tissue Type Collection (ATTC; Manassas, VA, USA) was cultured in DMEM medium supplemented with 100 U/mL penicillin, 100 µg/mL streptomycin and 10% fetal bovine serum in 75 cm^2^ tissue culture flasks at 37 °C in a humidified atmosphere of 5% CO_2_. Media exchange was performed every 3 days and cells were sub-cultured after having reached around 80% confluence. Glucose concentration was 20 mM according to ATTC suggestion.

H9c2 cells were transfected with a pIRES vector with or without exon(2-9)renin *cDNA* as previously described^8^. Upregulation of exon(2-9)renin mRNA was 10-fold as determined by qRT-PCR analysis. To ensure a steady overexpression of renin, the transfected pIRES control cells (empty vector) and the ren(2-9)-expressing cell line were cultured in the presence of 430 µg/mL G418 sulfate.

For functional analyses, pIRES control cells and exon(2-9)renin-transfected cells [ren(2-9)] were seeded in 6-well or 96-well culture plates, respectively, for 72 hours. They were then exposed to control conditions, glucose starvation, anoxia (AnaeroPack rectangular jar, Mitsubishi Gas Chemical Company Inc, Japan; GENbox anaer, Biomerieux, France) or the combination of oxygen and glucose depletion, for 24 hours at 5% CO_2_ and 37 °C, followed by qRT-PCR analyses for renin transcript abundance, detection of cell death, and analyses of mitochondrial parameters.

### Quantitative RT-PCR

RNA was extracted using the RNeasy Mini Kit (Zymo Research, Freiburg, Germany) according to the manufacturer’s instructions. Quality was checked by spectrophotometry (DS-11 + , DeNovix Inc, Wilmington, USA). High Capacity cDNA Kit (Life Technologies, Darmstadt, Germany) was used for reverse transcribing RNA to cDNA, which was stored at −70 °C. For qPCR, cDNA was diluted in nuclease-free water. Duplicates of 20 ng cDNA were mixed either with SYBR® FAST Universal 2X Master Mix containing SYBR green dye (Qiagen, Hilden, Germany) or with Blue 5′Green qPCR 2X Mix (Biozym, Hilden, Germany) and optimized primer pairs for the different renin transcripts and the housekeeping gene tyrosine 3-monooxygenase/tryptophan 5-monooxygenase activation protein, zeta (YWHAZ) (Table 1). The threshold cycle number (CT) in combination with the 2^−∆CT^ method was normalized against YWHAZ.

**Table 1:** Primer sequences for detection of transcript abundances.

Transcript	Forward primer	Revers primer
Renin exon(1-9)	ATGAATTCACCCCATTCAGC	CCAGATGGGCGGGAGGAGGATG
Renin exon(1A-9)	TGAATTTCCCCAGTCAGTGAT	GAATTCACCCCATTCAGCAC
Renin exon(2-9)	GCTCCTGGCAGATCACCAT	CCTGGCTACAGTTCACAACGTA
YWHAZ	CATCTGCAACGACGTACTGTCTCT	GACTGGTCCACAATTCCTTTCTTG
VEGF	TGCCAAGTGGTCCCAG	CGCACACCGCATTAGG
PDK1	CGGTGCCCCTGGCTGGATTT	GCATCCGTCCCGTAGCCCTC

### Western blot analysis

Proteins were extracted from trypsinated H9c2, pIRES and ren(2-9) cells using RIPA-lysis buffer containing 33.3 mmol/L Tris pH 7.5, 3.33 mmol/L EDTA, 100 mmol/L NaCl, 6.67 mmol/L K_2_HPO_4_, 6.7% glycerol, 0.67% Triton-X100, 0.03% SDS supplemented with 1 mmol/L Na_3_VO_4_, 20 mmol/L NaF, 0.1 mmol/L PMSF, 20 mmol/L 2-phosphoglycerate and a protease inhibitor cocktail (Roche Diagnostics, Mannheim, Germany). After sonification and determination of protein content (Roti-Quant Assay, Roth, Karlsruhe, Germany), a total of 25 µg of protein lysates were separated by SDS-PAGE under reducing conditions using 4-15% Criterion TGX gradient gels (BioRad Laboratories, Munich, Germany) and then transferred onto nitrocellulose membranes by a wet blot apparatus. For protein imaging, UV transillumination was performed using Chemidoc XRS (Bio-Rad Laboratories, Munich, Germany). Membranes were blocked with RotiBlock (Roth, Karlsruhe, Germany) for 1 h at room temperature (RT) followed by incubation with the primary rabbit anti-renin antibody (1:2000; Bioss Inc, Woburn, MA, USA) at 4 °C overnight and a further incubation with a horseradish peroxidase conjugated secondary anti-rabbit antibody (1:5.000; CellSignaling Technology, Leiden, The Netherlands). The protein expression was visualized by enhanced chemiluminescence method (BioRad Laboratories, Munich, Germany) and the image capture system (Chemidoc XRS, BioRad Laboratories, Munich, Germany). Whole protein was used as loading control, and the PageRuler Prestained Protein Ladder (Thermo Fisher Scientific Inc, Germany) served as molecular weight marker.

### Necrotic cell death

For determination of necrosis, 1 × 10^4^ cells in 100 µL medium were seeded in 96-well plates as six fold attempt. Three wells were used for the detection of spontaneous lactate dehydrogenase (LDH) release and the other 3 wells for the determination of the total cellular LDH content. After a 72-hours growing phase at 37 °C, 5% CO_2_ and incubation under ischemia-related conditions for another 24 h, necrosis rate was analyzed using the Cytotoxicity Detection Kit (LDH) (Roche Applied Science, Germany) as previously described^8^. For the detection of spontaneous LDH release, 100 µL of medium were added to 3 wells of the seeded cells, whereas in the other 3 wells 100 µl of 1% triton X-100 solution were given for 1 h to lyse the cells and record total LDH activity. Then, 100 µL of each well were transferred to another 96-well plate and incubated with 100 µL of the assay solution for 10 min in the dark. After measurements of absorbance at 490 nm, the necrosis rate was calculated by normalizing the amount of released LDH to the LDH content of the cells.

### Flow cytometry

For FACS analysis, control and treated adherent cells were detached from the 6-well plates by trypsin/EDTA treatment. Supernatants containing floating cells were collected and reunited with detached adherent cells. After centrifugation, cell pellets were re-suspended in culture medium. Cells were counted and apoptosis was determined by Annexin V (BD Pharmingen, Heidelberg, Germany), CaspACE FITC-VAD-FMK (Promega, Mannheim, Germany), and Fas receptor (Enzo Life Sciences, Lörrach, Germany) labelling according to the manufacturers’ instructions. Furthermore, levels of reactive oxygen species (ROS) were analyzed using the ROS-sensitive fluorophores MitoSOX and dihydroethidium (DHE), while free cytosolic Ca^2+^ content was detected by the fluorophore Fluo3AM.

Briefly, 10^5^ cells were incubated with either 5 µL Annexin V-FITC dye in 100 µL Annexin binding buffer for 15 min at room temperature in the dark, with 5 µL of the 1:100 diluted CaspACE FITC-VAD-FMK in 100 µL culture medium for 20 min at 37 °C or with 1.5 µL primary rabbit anti-CD95 antibody (Fas receptor) in 100 µL FACS buffer for 15 min at 4 °C. The unbound antibodies or dyes were removed by washing the cells with 3 mL Annexin binding buffer or with FACS buffer. Regarding Fas receptor expression, a second incubation with 10 µL of 1:10 diluted anti-rabbit IgG-FITC antibody was performed for 15 min at 4 °C. Again, the unbound antibody was washed out with 3 mL FACS buffer. Before measurement, cells were additionally incubated with 500 ng/mL propidium iodide (PI) for 5 min to discriminate between early and late apoptotic as well as early necrotic cells. PI labelling combined with the use of apoptotic markers allows the discrimination between necrotic and apoptotic cells. Necrotic cells, which showed PI but non apoptosis staining, were in an early state of necrosis with disrupted membrane integrity but still preserved cellular integrity.

For analyzing ROS, 10^5^ cells were incubated at 37 °C in 500 µL culture medium supplemented with either 5 µL MitoSOX Red mitochondrial superoxide indicator (5 µmol/L, Invitrogen, Molecular Probes) for 30 min or with 10 µL DHE (2 µmol/L, Invitrogen, Molecular Probes) for 30 min. After washing in 2 mL FACS buffer, cells were re-suspended in 500 µL medium and analyzed.

Cytosolic free Ca^2+^ content was determined in 1 × 10^5^ cells that were incubated for 45 min at room temperature in 500 µL RPMI 1640 medium plus 2% FCS and 2 mmol/L HEPES, supplemented with 1 µL Fluo3AM (2 µmol/L, Biotrend) / Pluronic F127 mixture (1:1). After washing in 2 mL Hanks buffer, cells were re-suspended in 500 µL Hanks buffer, incubated for another 30 min at room temperature in the dark and then analyzed.

Data from 5.000 cells were analyzed on a FACS Calibur flow cytometer (BD, Franklin Lakes, NJ, USA). Cell debris was excluded from the measurement by setting a gate for intact cells. The data were analyzed by Cell Quest software (BD Biosciences, Franklin Lakes, NJ, USA).

### Imaging mitochondrial parameters

Cells (10^4^/well) were grown in 96-well plates for 72 hours in a humidified atmosphere (5% CO_2_ at 37 °C) to allow attachment and adaptation. Then, cells were exposed to control or ischemic conditions for another 24 hours before analyzing ATP levels. Because OGD was accompanied by severe necrosis, we analyzed ATP levels in cells and in the supernatant, after centrifugation of the plates. For measurements of ATP content, the CellTiter-Glo® Luminescent Cell Viability Assay was used according to manufacturer’s instructions (Promega, Mannheim, Germany). This assay is based on the conversion of luciferin to oxiluciferin, pyrophosphate and light in the presence of ATP. The quantity of light was measured using a microplate luminometer (Berthold, Bad Wildbach, Germany).

Mitochondrial membrane potential (∆Ψ_m_) was measured using 5,5′,6,6′-tetrachloro-1,1′,3,3′-tetramethylbenzimidazolylcarbo-cyanine iodide (JC1) according to manufacturer’s instructions (Molecular Probes, Thermo Fisher Scientific, Germany). 1 × 10^5^ cells in 0.5 mL culture medium were incubated with 50 µL of JC1 solution (4 µmol/L) for 15 min at 37 °C in the CO_2_ incubator. For JC1 excitation a 488 nm filter was used. Emission filters of 535 nm and 595 nm were used to quantify cells with green (JC1 monomers) and orange (JC1 aggregates) fluorescence, respectively. The ratio of orange/green fluorescence intensity reflects the mitochondrial membrane potential.

In addition to JC1, the calcein-AM/cobalt chloride (CoCl_2_) method (MitoProbe™ Transition Pore Assay Kit, Molecular Probes, Invitrogen) was used for detection of the opening of the mitochondrial permeability transition pore (mPTP) according to the manufacturer’s instructions. Briefly, 1 × 10^5^ pretreated pIRES or ren(2-9)cells were incubated with 2.0 µmol/L calceinAM in HBSS/Ca^2+^ buffer for 15 min at 37 °C in duplicates. Calcein-AM passively diffuses into the cells and accumulates in the cytosol and mitochondria. In one vial, the medium was additionally supplemented with 80 mmol/L cobalt chloride to quench the cytosolic calcein fluorescence, while the fluorescence of mitochondrial calcein is maintained. After incubation, excessive calcein was washed out by addition of 2 mL HBSS/Ca^2+^ buffer and centrifugation of the cells. Cells were then re-suspended in HBSS/Ca^2+^ buffer and analyzed on a FACS Calibur flow cytometer using Cell Quest software.

### Statistical analyses

The data presented are individual and means ± SEM of independently performed experiments. To specify differences between groups, one-way and two-way ANOVA with Bonferroni posttest analyses, or ANOVA on ranks (Kruscal Wallis), in cases, where the requirements of ANOVA were not fulfilled (unequal variances), were performed as appropriate using GraphPad Prism (Graph Pad Software, La Jolla California, USA). Values of p < 0.05 were considered statistically significant.

## Supplementary information

Dataset 1.
