# Supplementary material for: Non-secretory renin reduces oxidative stress and increases cardiomyoblast survival during glucose and oxygen deprivation
Source: Sci Rep. 2020 Feb 11;10:2329. doi: 10.1038/s41598-020-59216-8 (PMC7012910; doi:10.1038/s41598-020-59216-8)
Supplement: Supplementary file 1 — Dataset 1. [file 41598_2020_59216_MOESM1_ESM.pdf]

Non-secretory renin reduces oxidative stress and increases cardiomyoblast survival during glucose and oxygen deprivation

Heike Wanka, Philipp Lutze, Doreen Staar, Alexander Albers, Inga Bäumgen, Bianka Grunow & Jörg Peters

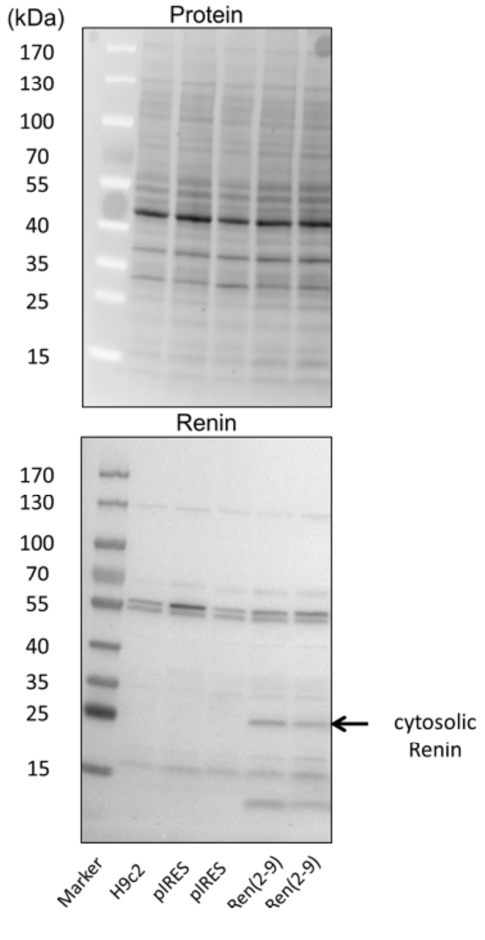

Suppl. 1. Expression of renin transcripts in transfected pIRES and ren(2-9) cells. Renin protein of pIRES controls and ren(2-9) cells normalized to the protein content was detected by Western Blot.

For protein imaging, UV transillumination was performed using Chemidoc XRS (Bio-Rad Laboratories, Munich, Germany). The protein expression was visualized by enhanced chemiluminescence method (BioRad Laboratories, Munich, Germany) and the image capture system (Chemidoc XRS, BioRad Laboratories, Munich, Germany). Whole protein was used as loading control, and the PageRuler Prestained Protein Ladder (Thermo Fisher Scientific Inc, Germany) served as molecular weight marker.
